# A new approach for incorporating sea-level rise in hybrid 2D/one-line shoreline models

**DOI:** 10.1038/s41598-022-23043-w

**Published:** 2022-10-27

**Authors:** Avidesh Seenath

**Affiliations:** Faculty of Engineering, Environment and Computing, Coventry University, Coventry, CV1 2LT UK

**Keywords:** Climate and Earth system modelling, Projection and prediction, Geomorphology

## Abstract

Hybrid 2D/one-line shoreline models, which typically apply a finite volume approach to simulate sediment transport and the one-line theory to update the shoreline morphology, are being increasingly applied over meso timescales (10^1^ to 10^2^ years) to inform coastal management. The one-line theory assumption of a constant closure depth prevents these models from considering the effects of sea-level rise in the shoreline morphology update. Sea-level rise, an endogenous driving factor of meso timescale coastal behaviour, influences the closure depth through its effects on the wave climate. This paper presents a new hybrid 2D/one-line approach that enables a time-varying closure depth in response to annual variations in wave climate as a solution for mirroring the effects of sea-level rise on the coastal profile and associated shoreline evolution. This new hybrid approach is applied to hindcast meso timescale shoreline evolution in a sandy coastal system and compared against the traditional hybrid 2D/one-line approach. Results show that the traditional hybrid approach gives the most accurate predictions whereas the new hybrid approach overpredicts shoreline erosion. However, this overprediction is attributed to net closure depth overestimation. This attribution gives confidence that the shoreline response to the time-varying closure depth specified is within expectations since closure depth overestimation increases offshore sediment transport in shoreline models. Therefore, it is likely that enabling a time-varying closure depth in hybrid 2D/one-line models may improve meso timescale shoreline predictions under sea-level rise if closure depths can be accurately prescribed over time.

## Introduction

There is an increasing demand for meso timescale (10^1^ to 10^2^ years) shoreline evolution models that account for the combined effects of sea-level rise and coastal engineering to inform coastal management^1–5^, particularly in sandy coastal systems in vulnerable small islands^6^. The morphology of these systems continually adjusts in response to subtle changes in external forcings, such as wave-climate, sea-level rise, and coastal engineering (Table 1)^4,7,8^. Sandy coastal systems are subject to significant human interferences globally (e.g. development, coastal engineering, tourism) and are the primary socioeconomic resource for many small island states^9,10^. Human interferences and 20th-century sea-level rise have accelerated the erosion of sandy shorelines in many regions, resulting in the widespread use of coastal engineering, such as hard defences (e.g. groynes and sea walls) and beach nourishment^11,12^. The widespread use of hard defences, in particular, threatens the continued existence of sandy coastal systems by limiting their ability to migrate under sea-level rise^13^. Hard defences reduce erosion by deflecting wave energy, shifting the erosion problem downdrift^14^. Downdrift erosion from hard defences is a critical problem affecting sandy coastal systems in many small island states in the Caribbean and Pacific^10,15^. These islands are projected to be severely challenged by sea-level rise this century^16^. Sea-level rise will likely worsen the erosion of managed sandy shorelines by modifying wave climates, and the resulting interactions between wave-generated currents and hard defences^6,17^. The combined effects of sea-level rise and hard defences on the evolution of sandy coastal systems are likely to manifest over meso timescales^14,18,19^.

**Table 1:** External forcings associated with each established scale of shoreline evolution (adapted from^20,51^).

Scale	Natural forcing	Human forcing
Macro Space dimensions: ≥ 100 km Time dimensions: centuries to millennia	Sediment availability Relative sea-level changes Differential bottom changes Geological setting Long-term climate changes Paleomorphology (inherited morphology)	Human-induced climate change Major river regulation Major coastal structures Major reclamations and closure Structural coastal (non)management
Meso Space dimensions: ~ 10 to 100 km Time dimensions: decades to centuries	Relative sea-level changes Regional climate variations Coastal inlet cycles Sand waves Extreme events	River regulation Coastal structures Reclamations and closures Coastal (non)management Natural resource extraction (subsidence)
Synoptic Space dimensions: ~ 1 to 5 km Time dimensions: years to decades	Wave climate variations Surf zone bar cycles Extreme events	Surf zone structures Shore nourishments
Micro Space dimensions: ~ 10 m to 1 km Time dimensions: hours to years	Wave, tide, and surge conditions Seasonal climate variations	

Coastal management is typically informed by shoreline evolution predictions from two-dimensional horizontal (2DH) or behaviour-oriented models^21–23^. 2DH models simulate the physics of shoreline evolution on a horizontal plane, incorporating the combined effects of various external forcings, including sea-level rise and coastal engineering^24^. As these models are discretised on a horizontal plane, they cannot account for the vertical variations of undertow currents, which strongly influence surf zone morphodynamics^25,26^. Undertow currents are seaward directed currents that move beneath the surface of waves approaching the shoreline. These currents are the primary driving flux of cross-shore sediment transport, and their interactions with waves influences the evolution of coastal profiles^25,27,28^. An inability to simulate the vertical variations of undertow currents means that 2DH models cannot describe the delicate balance of the cross-shore sediment transport that evolves the coastal profile^29^. As a result, the coastal profile gradually degenerates to an erroneous shape in these models^30^. The gradual degeneration of coastal profiles introduces errors at each time step in a simulation, causing shoreline evolution predictions to become unreliable in simulations longer than micro timescales (< 10^1^ years)^26,29,31,32^. These limitations of 2DH models have encouraged the application of behaviour-oriented models for simulating meso timescale shoreline evolution^8,24,33^.

Behaviour-oriented models are simple mathematical formulations that simulate known coastal behaviours rather than the physics underlying the known coastal behaviours^5,21,34^. These models use a rule-based approach and a diffusion type formulation that force the evolution of active coastal profiles towards an equilibrium form. The active coastal profile extends from the beach berm to closure depth. The closure depth is generally regarded as the depth limit of significant wave action, which is also the depth beyond which there is no significant sediment transport^35^. Behaviour-oriented models focus on processes that drive shoreline evolution over decades to centuries and consider processes operating at smaller timescales as noise^23,36–39^. The one-line theory and the Bruun Rule typically form the basis of behaviour-oriented models. The one-line theory assumes the active coastal profile moves shore-normal in response to littoral drift gradients, whereas the Bruun Rule assumes the active coastal profile shifts upward and landward from sea-level rise^40,41^. The generalisation of the underlying physics in model calculations and the assumption of an equilibrium coastal profile ensure a stable shoreline morphology update over meso timescales, but prevent behaviour-oriented models from simulating the combined effects of sea-level rise and coastal engineering on shoreline evolution^22^.

The constraints of 2DH and behaviour-oriented models have inspired the development of hybrid 2D/one-line models for simulating meso timescale shoreline evolution^30,42,43^. Hybrid models maintain the physics-driven approach of 2DH models but apply the one-line theory to update the shoreline morphology^44^. They simulate the physics of coastal sediment transport on a horizontal plane, incorporating the combined effects of sea-level rise, coastal engineering and other external forcings, and uniformly redistribute the derived littoral drift gradients over the active coastal profile. The active coastal profile moves shore-normal from a change in sediment balance, resulting in a change in shoreline position^25,29^. Using the one-line theory to update the shoreline morphology prevents the erroneous breakdown of coastal profiles, allowing hybrid 2D/one-line models to simulate meso timescale shoreline evolution whilst accounting for the physics of coastal sediment transport^29^. However, the use of the one-line theory means that hybrid 2D/one-line models cannot account for sea-level rise in the shoreline morphology update as the one-line theory assumes the active coastal profile maintains a constant time-averaged equilibrium form, which implicitly implies that the closure depth remains constant^30^. Sea-level rise is known to change the closure depth through its effects on the wave climate and will likely be an endogenous driver of coastal evolution over meso timescales^45,46^. Therefore, accounting for sea-level rise in meso timescale shoreline evolution simulations is currently a novel scientific challenge.

Sea-level rise will inevitably modify the wave setup and undertow currents by forcing deeper waters closer to the shoreline^25,47,48^. An increase in water depth will cause wave breaking closer to the shoreline, potentially increasing the undertow mobilisation and offshore transport capacity of near-bed sediments^47,49^. Consequently, sea-level rise implicitly modifies the shape of the active coastal profile, particularly the offshore part, by affecting the mass balance of cross-shore sediment transport through its effects on the wave setup and associated undertow^47,48^. A notable effect of wave setup variations is a change in the closure depth, which represents the depth limit of significant wave action^50^. Changes in closure depth affect the shoreface morphology by influencing the cross-shore extent of morphodynamics^51^. The closure depth is, therefore, a good proxy of wave climate and sea-level change^52,53^. Allowing the closure depth to vary over time in hybrid 2D/one-line models may, thus, enable us to mirror the effects of sea-level rise on the offshore part of the active coastal profile^51^. Doing so (a) would not compromise the stability of the shoreline morphology update as the underlying principles of the equilibrium coastal profile concept would be maintained; and (b) may offer an interim novel solution to account for sea-level rise in meso timescale shoreline evolution simulations until we can effectively account for undertow currents in these simulations.

Considering the above arguments, this paper applies an experimental numerical modelling study to assess (a) the validity of the one-line theory for simulating shoreline evolution under sea-level rise, and (b) whether we can obtain more theoretically realistic predictions of meso timescale shoreline evolution under sea-level rise by allowing the closure depth to vary temporally in hybrid 2D/one-line models.

## Test site

**Figure 1:**
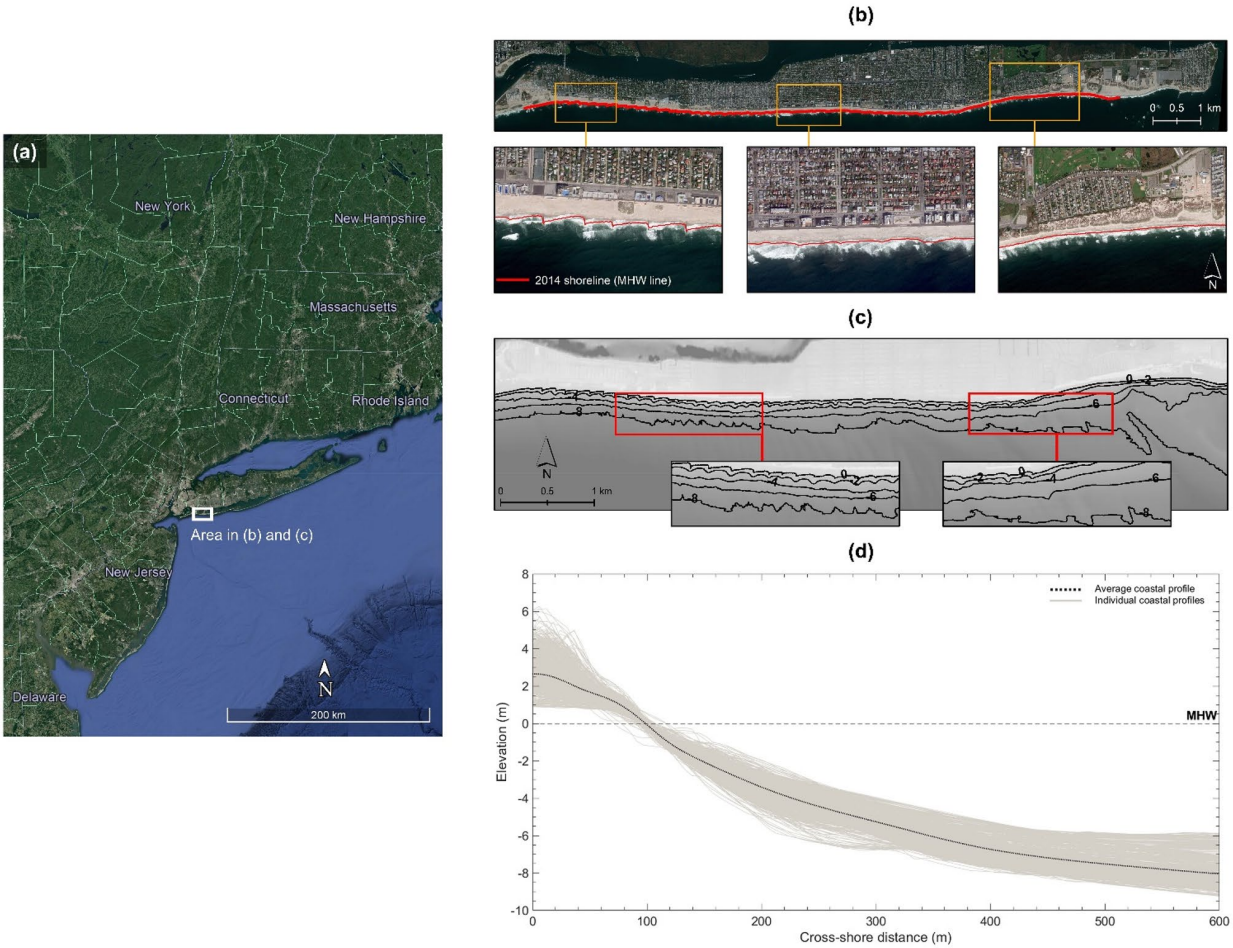
Test site in New York (adapted from^51^). (**a**) Location along the United States East Coast. (**b**) 2012 GeoEye-1 image of the main site features. (**c**) Contour map illustrating shore-parallel depth contours in the nearshore. (**d**) 2014 coastal profile envelope and average coastal profile. *Credits*: Google Earth (satellite image in a) and LAND INFO Worldwide Mapping (GeoEye-1 image in **b**).

A 12.5 km sandy coastal stretch, located along the Atlantic coast of Long Beach Barrier Island (Fig. 1), is selected to address the aims of this paper. The shoreline here, defined as the Mean High Water (MHW) line, is managed by 43 groynes. It is concave in the east and west and generally straight elsewhere, except for deformations from accretion (erosion) updrift (downdrift) of the groynes (Fig. 1b). The area is microtidal (mean tide range = 1.43 m) and has a simple planform morphology, defined by shore-parallel depth contours (Fig. 1c). The average coastal profile gently slopes and decreases monotonically cross-shore, reflecting the envelope of coastal profiles sampled every 15 m longshore from a 2014 Digital Elevation Model (DEM) (Fig. 1d).

This test site is selected for two reasons. *First*, its simple planform morphology conforms to the one-line theory assumptions of shore-parallel depth contours, which underpin the shoreline morphology update in hybrid 2D/one-line models. The site thus provides an appropriate basis for developing and testing a new hybrid 2D/one-line approach that accommodates a time-varying closure depth as a solution for incorporating sea-level rise effects in shoreline evolution simulations in order to address the aims of this paper. Note that it is not the intent of this paper to provide a case study analysis of shoreline change in the test site selected but rather use the test site solely as a basis for continuing the development of the hybrid 2D/one-line modelling approach in order to address its present limitations. The results generated at the test site should, therefore, be interpreted primarily in light of the modelling approaches tested in this paper. The *second* reason for selecting the above test site is data availability. The test site selected has extensive open-source high-resolution coastal data (tides, wind, wave climate, and DEMs) (Table 2), which is an essential requirement for the development and testing of all new modelling approaches.

**Table 2:** Data for model calibration (2014–2016) and meso timescale hindcasts (1966–2016).

Data	Time period	Application	Horizontal datum	Vertical datum	Units	Resolution	Source
Initial bathymetry	01-01-2014 01-01-1966	Calibration Meso timescale hindcasts	WGS84	MHW	m	3 m 10 m	^56^ ^55^
Observed bathymetry	01-02-2016	Calibration Meso timescale hindcasts				3 m	^57^
Tide	01-01-2014–01-02-2016 01-01-1966–01-02-2016	Calibration	Not applicable			6 min	^58^
		Meso timescale hindcasts				60 min	
Wind speed	01-01-2014–01-02-2016	Calibration Meso timescale hindcasts		Not applicable	m/s	6 min	
Wind direction					deg		
Wave height					m	60 min	^59^
Wave direction					deg	60 min	
Wave period					s	60 min	

## Data and methods

### Study periods and data

Considering the high-resolution DEMs and coastal processes data available for Long Beach Barrier Island (Table 2), a micro and meso timescale period are defined as follows: (a) 2014–2016 for model calibration and (b) 1966–2016 for meso timescale applications. A micro timescale period is deemed sufficient for model calibration because if a model fails to predict realistic shoreline evolution over this timescale, the uncertainties introduced will propagate and generate unreliable predictions over meso timescales^51^. Also, rates and patterns of meso timescale shoreline evolution are influenced by local environmental factors and processes that operate at the micro timescale, such as waves, supra-, inter-, and sub-tidal morphology, and sedimentology^54,55^. Therefore, evaluating the ability of mesoscale shoreline evolution models to provide realistic micro timescale predictions is a necessary first step towards identifying (and addressing) their key limitations before applying them over meso timescales.

A 1966^56^, 2014^57^, and 2016^58^ DEM of Long Beach Barrier Island are obtained. These are all vertically referenced to MHW (m) and horizontally referenced to WGS 84 (m). The MHW line in each DEM (i.e. the zero-depth contour) is considered to be the shoreline position. The 2014 and 2016 DEMs have a spatial resolution of 3 m, and the 1966 DEM has a spatial resolution of 10 m. The 2014 DEM contains topobathymetric data surveyed at the start of the model calibration period and, therefore, provides the initial bed morphology for the model calibration simulations of shoreline evolution. The 1966 DEM contains topobathymetric data surveyed at the start of the meso timescale study period and, therefore, provides the initial bed morphology for the meso timescale simulations of shoreline evolution. The model calibration and meso timescale study periods both end in 2016, corresponding to the period of the topobathymetric survey that make up the 2016 DEM. The shoreline in the 2016 DEM is thus used as the benchmark for quantifying the accuracy of shoreline evolution predictions from the model calibration and meso timescale simulations.

Table 2 summarises the metadata of the tide^59^, wind^59^, and wave-climate^60^ data obtained for model calibration and meso timescale simulations of shoreline evolution. All tide data are vertically referenced to MHW (m). All wind data include wind speed (m/s) and direction (deg.), and all wave climate data include wave height (m), period (s), and direction (deg.).

A 2012 georeferenced GeoEye-1 satellite image (spatial resolution = 0.5 m) of Long Beach Barrier Island is used to extract and obtain data on the location and geometry of all groynes present at the test site. Data on the elevation of the groynes are derived from the DEMs obtained.

### Model selection

Considering Seenath^51^ review of the existing capabilities of shoreline evolution models, MIKE21 is selected to address the aims of this paper for three reasons:

*First*, unlike related hybrid models, MIKE21 links a 2DH coupled wave, flow, and sediment transport model, which is discretised on a finite volume mesh, with a one-line model of shoreline change. This enables meso timescale simulations of littoral drift that account for the combined effects of external forcings (e.g. waves, tides, sea-level rise, and hard defences) across simple and complex morphologies^61^. This capability is necessary for considering the effects of groynes on littoral drift gradients, which affects shoreline evolution in the test site^62–64^. Linking the 2DH sediment transport modelling approach with a one-line shoreline model prevents the erroneous breakdown of coastal profiles, which restricts physics-driven models (e.g. 2DH models) to micro timescales, and enables meso timescale applications^29^.

*Second*, MIKE21 uses local coordinates in the one-line shoreline model, allowing each point along a shoreline to evolve perpendicular to its orientation^61^. This allows MIKE21 to handle complex shoreline geometries, including the curvature and deformations that characterise the shoreline in the test site.

*Third*, MIKE21 is currently the only known hybrid 2D/one-line model that accounts for both (a) complex planform morphologies (i.e. non-parallel depth contours) in littoral drift simulations and (b) shoreline deformations and curvature in shoreline evolution predictions^51^. It, therefore, provides a good basis for continuing the development and refinement of the hybrid 2D/one-line modelling approach.

### Model description

**Figure 2:**
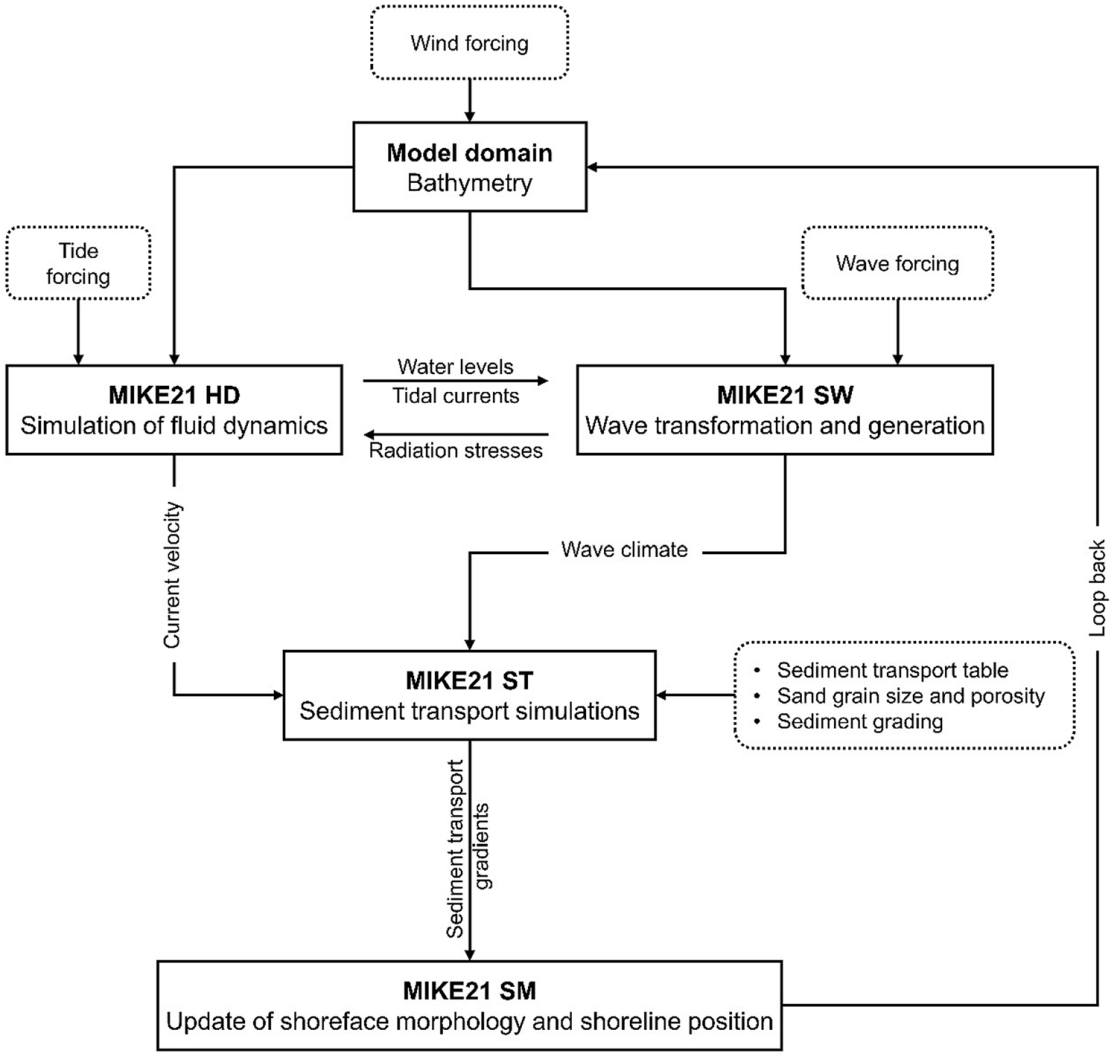
Computational framework of MIKE21^51^. MIKE21 SW and MIKE21 HD simulate the wave and flow field on a finite volume mesh, respectively. MIKE21 ST simulates the littoral drift gradients in response to the wave and flow fields, and MIKE21 SM uses the littoral drift gradients to update the shoreline position at each time-step based on the one-line theory (Fig. 3).

MIKE21 combines a two-dimensional description of waves, hydrodynamics, and sediment transport with a one-line description of the shoreline position by coupling four modules (Fig. 2): Spectral Wave (MIKE21 SW), Hydrodynamic (MIKE21 HD), Sand Transport (MIKE21 ST), and Shoreline Morphology (MIKE21 SM). MIKE21 is a commercial code with extensive documentation on its computational structure^6,61^. However, a brief overview of MIKE21 ST and MIKE21 SM is provided below since MIKE21 ST generates the littoral drift gradients that provide the primary forcing in MIKE21 SM, which forms the basis for the continued development of the hybrid 2D/one-line approach in this paper. For an extensive description of all modules, please see DHI^61^.

MIKE21 ST simulates sediment transport from combined wave-current action^65^. This module provides a quasi-3D description of the force balance and hydrodynamics through the water column and a detailed description of the instantaneous turbulent boundary stresses from wave-current interactions. Fredsøe^66^ integrated momentum approach calculates the time and vertical variations in bed shear stress, turbulence, flow velocity, and sediment concentration. MIKE21 ST determines total sediment transport by calculating bed load and suspended load transport separately. The bed load transport is derived from the instantaneous Shields parameter using Engelund and Fredsøe^67^ model, and the suspended load transport is the product of the instantaneous flow velocities and sediment concentration. Vertical variations in suspended sediment concentration are derived from Fredsøe et al.^68^ vertical diffusion equation for suspended sediment. In a simulation, MIKE21 ST calculates sediment transport rates by linear interpolation in a precomputed sediment transport table based on the wave, current, and water level conditions in MIKE21 SW and MIKE21 HD. The sediment transport table considers the range of wave, current, and sediment (grain size and sediment sorting) conditions that are likely to occur in the simulation. The littoral drift gradients calculated from MIKE21 ST update the shoreface morphology and shoreline position according to MIKE21 SM shoreline continuity equation. For further details on this module, please see DHI^65^.

**Figure 3:**
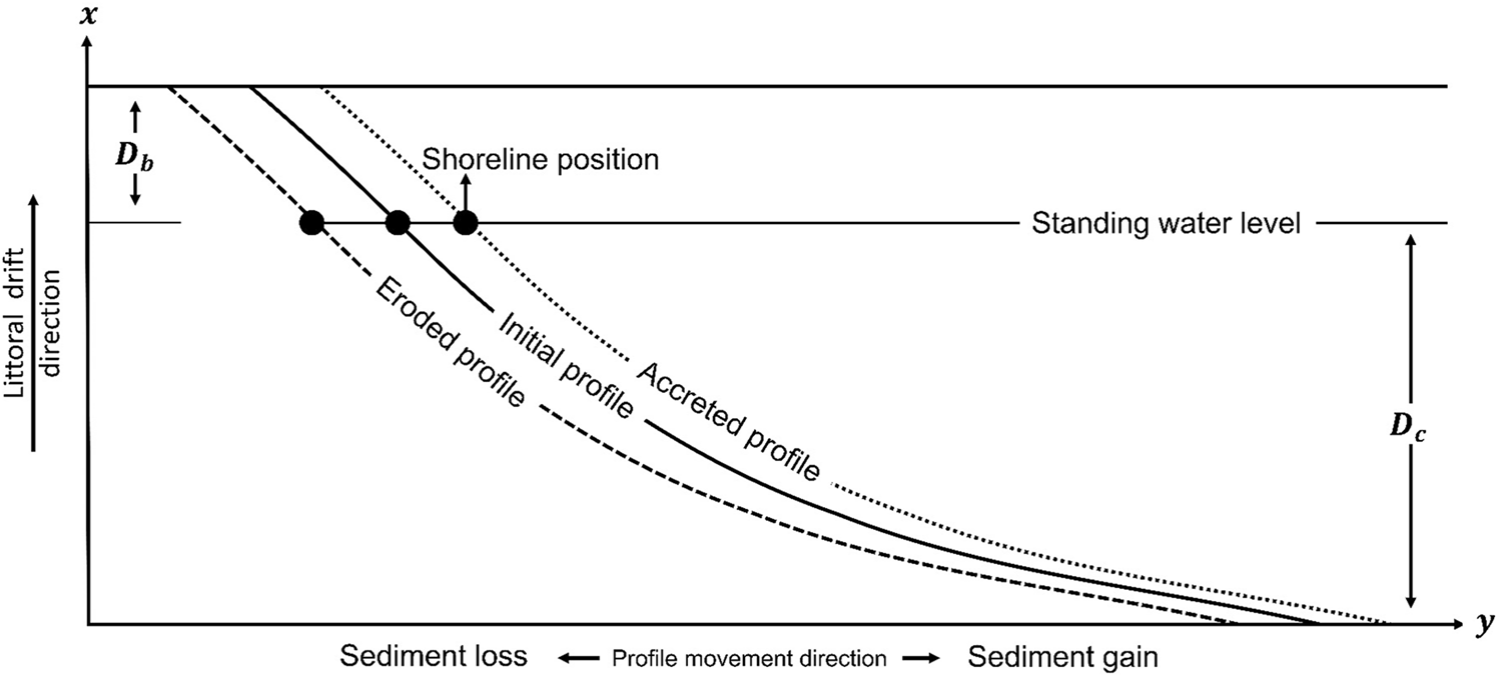
One-line approach used for simulating shoreline change in hybrid 2D/one-line models, such as MIKE21^51^. Shoreline change is a function of the shore-normal movement of the active coastal profile (beach berm—${D}_{b}$—to closure depth—${D}_{c}$). A gain (loss) in sediment shifts the active coastal profile seaward (landward).

MIKE21 SM applies the one-line theory to simulate changes in shoreline position based on littoral drift gradients calculated from MIKE21 ST (Fig. 3). It divides the shoreface into strips perpendicular to the shoreline. At each time step in a simulation ($\Delta t$), MIKE21 SM integrates the change in sediment volume ($vol$) within each shoreface strip with a predefined active coastal profile (beach berm to closure depth) to calculate the change in shoreline position using a modified version of the one-line theory equation:

1 $$\frac{\Delta N}{\Delta t}= \frac{vol}{{dA}_{z}}$$

where $\Delta N$ is the horizontal distance over which the shoreline moves shore-normal, and ${dA}_{z}$ is the vertical area of the active coastal profile within each shoreface strip over which $vol$ is uniformly distributed. The change in sediment volume at each time step is determined from the littoral drift gradients calculated in MIKE21 ST. The active coastal profile shifts seaward in response to sediment gain and landward in response to sediment loss in accordance with the one-line theory (Fig. 3).

### Mesh generation

The spatial domain for generating the computational mesh to apply MIKE21 is defined in UTM coordinates with a cross-shore dimension of 2 km and a longshore dimension of 12.5 km. The domain encompasses all of the land area in the test site and extends seaward to a depth of approximately 13 m below MHW. The mean wave height in the wave data obtained (Table 2) is 1.2 m (standard deviation = 0.69). A 13 m depth boundary is, therefore, adequately deep to not affect wave propagation and wave approach to the shoreline as waves normally break at a depth equivalent to 1.2 times their height.

**Figure 4:**
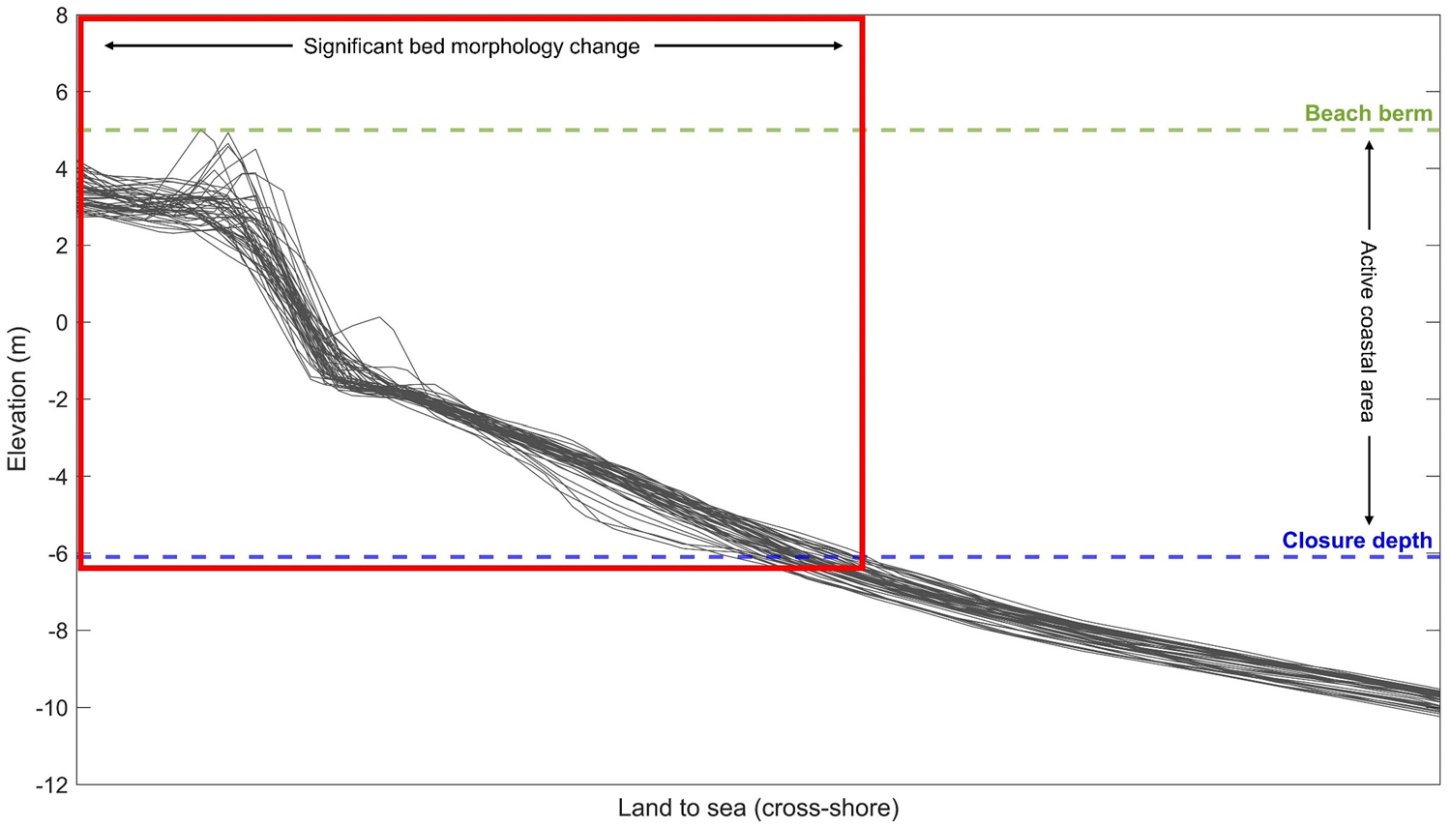
Schematic illustration on identifying the closure depth along a coastal profile. The closure depth is traditionally considered to be the depth beyond which we can see no significant changes in bottom elevation (bed morphology). This typically coincides with the basal limit of the envelope of coastal profile changes.

The spatial domain is divided into two zones, nearshore and offshore, each separated by the closure depth. There are several qualitative indicators that are commonly used to identify the closure depth in simple planform morphologies. Historically, the closure depth is considered to be the most landward depth seaward of which we can see no significant change in bottom elevation^69^. This depth corresponds to the point along a beach profile where we start to see the profile slowly becoming steeper (a second indicator) and is easily identifiable by (a) the base of the envelope of coastal profile changes (Fig. 4)^70,71^ or (b) the depth contour that mirrors the shoreline shape^30^. Related studies on the continued development of the hybrid 2D/one-line modelling approach have considered the depth contour that follows the shoreline shape as the closure depth^30^. The key reason for this is linked to the one-line theory assumptions, which underpin the shoreline morphology update, in hybrid 2D/one-line models^29,30^. The one-line theory implicitly implies that all contour lines have a similar time-averaged shape and simply move landward and seaward up to the closure depth as if there were only one contour line^41^. Thus, as per the one-line theory, the closure depth is the most seaward depth contour that reflects the shoreline shape. This closure depth indicator is, therefore, adopted here to initially setup the model for continuing the development of the hybrid 2D/one-line modelling approach.

**Figure 5:**
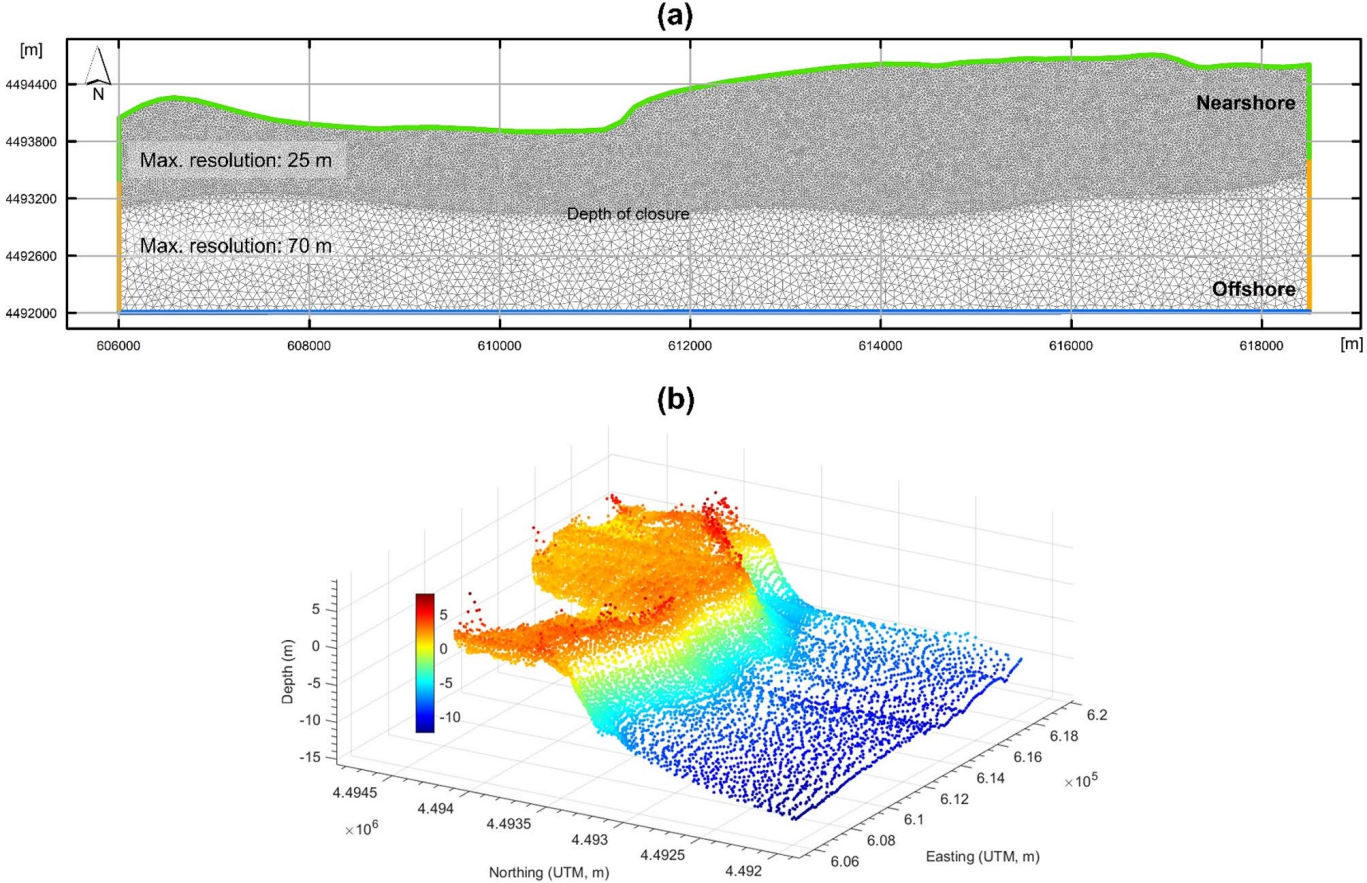
Computational mesh used for shoreline evolution simulations. (**a**) Finite volume mesh generated. (**b**) Mesh nodes interpolated with topography and bathymetry data from the 2014 DEM obtained.

A maximum element area of 625 m^2^ (25 m resolution) is specified nearshore and 4,900 m^2^ (70 m resolution) offshore to generate the mesh in the spatial domain using Shewchuk^72^ Delaunay refinement method. These resolutions create the finest mesh discretisation that both (a) enables numerical convergence and (b) is computationally feasible to apply MIKE21 in the test site. Importantly, the nearshore and offshore resolutions defined correspond to the spatial scales over which the primary drivers of shoreline evolution (e.g. waves) operate (Table 1). The resulting mesh is a finite volume discretisation, with 42,154 triangular elements and four boundaries: land, sea, and two connecting (Fig. 5).

Using the natural neighbour approach, (a) the 2014 DEM obtained is interpolated onto the mesh to generate the initial mesh bathymetry for model calibration simulations of shoreline evolution, and (b) the 1966 DEM obtained is interpolated onto the mesh to generate the initial mesh bathymetry for the meso timescale hindcast simulations of shoreline evolution. The natural neighbour approach creates a triangulated irregular network from the $x,y,z$ data points in the DEM and assigns a weighted value to each data point surrounding each mesh node based on the distance from the data point to the node. The depth in the centre of a triangular mesh element is the average of the depth values interpolated at each of the element nodes. The natural neighbour approach is selected because it preserves the topobathymetric data and the associated coastal profile morphology from the source DEMs. It is important that observed coastal profile characteristics are well-captured in the computational mesh otherwise there is an increased risk of obtaining unreliable shoreline evolution predictions that can compromise decisions on (a) model development and refinement and (b) coastal zone management.

### Model parameterisation

#### Boundary conditions

The high-resolution tide and wave data obtained are forced at the sea boundary in the mesh and the connecting boundaries are kept open to facilitate littoral drift. The effects of wind on waves and currents are included by forcing the high-resolution wind data obtained over the model domain. All forcings are entered with a dampened interval of two hours to prevent shock waves inside the model domain.

A zero-sediment flux gradient is specified at all mesh boundaries, excluding the land boundary, to ensure mass conservation and prevent instabilities from generating at the boundaries. A zero-sediment flux gradient is an open boundary condition that allows the same volume of sand in and out of the boundaries as demanded by the changing wave-current conditions in the model domain^51^. This type of boundary condition prevents the sudden deposition or erosion of sediment at the open boundaries^73^, which can destabilise model solutions and prevent mass conservation.

Each module in MIKE21 is parameterised, as outlined in Table 3, to account for key coastal system characteristics that are not adequately represented by the boundary conditions specified. All specifications in Table 3 are based on established guidelines for setting up coastal morphology models for application in sandy coastal systems^61,74^.

**Table 3:** Initial (pre-calibration) specifications used to set up MIKE21 for application in the test site.

Input	Specifications
**General**	
Simulation period (model calibration only)	01-Jan-2014 to 01-Feb-2016
Time step interval (output frequency)	86,400 s (daily)
**MIKE21 HD**	
Coriolis forcing	Varying in domain
Courant–Friedrich–Lévy (CFL) number	0.8
Density	Barotropic
Manning’s *n* reciprocal^a^	
	32 m^1/3^/s
Maximum time step	30 s
Minimum time step	0.01 s
Overtopping discharge	0 m^3^/s/m
Smagorinsky coefficient (eddy viscosity)	0.28
Wave radiation stresses	Internally transfers from MIKE21 SW
Weir coefficient^a^	1.838 m^1/2^/s
Wind forcing	Wind speed and direction data
Wind friction (varies based on wind speed)	0.001255 to 0.002425
**MIKE21 ST**	
Critical shields parameter	0.05
Grading coefficient^a^	1.1
Grain diameter^a^	0.2 mm
Flow/wave forcing	Internally transfers from MIKE21 SW
Maximum bed level change	10 m/day
Porosity^a^	0.4
Relative sand density	2.65
Time step factor	1
**MIKE21 SW**	
Current conditions (speed and direction)	Internally transfers from MIKE21 HD
Maximum number of iterations	500
Nikuradse roughness	0.04 m
Reflection coefficient (structures)	0.5
Spectral discretisation	360 degree rose
Water level conditions	Internally transfers from MIKE21 HD
**MIKE21 SM**	
Berm height	1.14 m
Closure depth	5.8 m
Maximum number of iterations	500
Littoral drift gradients	Internally transfers from MIKE21 ST

^a^Calibration parameter.

MIKE21 ST estimates littoral drift rates by linear interpolation in a precomputed sediment transport table based on hydro-morphodynamic conditions operating in the model domain^65^. This table defines the range of current speed, wave height, wave period, wave height to water depth ratio, the angle between current and waves, median grain size, sediment grading, and bed slope that is likely to appear in the simulation. MIKE21 ST calculations of littoral drift rates become unreliable if these conditions in a simulation do not fall within their defined range in the precomputed sediment transport table, which generates uncertainty in associated shoreline evolution predictions. Following Manson^74^, a stepwise calibration approach is used to establish the precomputed sediment transport table (Table 4) that best describes the hydro-morphodynamic conditions at the test site. This calibration process takes into account the coastal data obtained as well as observed morphological conditions at the site^62,64^.

**Table 4:** Calibrated sediment transport table for shoreline evolution simulations.

Sediment table axis	First value	Spacing	No. of points
Current speed (m/s)	0.01	0.8	5
Wave height (m)	0.19	2	4
Wave period (s)	2.35	2	8
Wave height to water depth ratio	0.01	10	10
Angle between current and waves (deg)	0	30	12
Median grain size (mm)	0.2	2	8
Sediment grading	1.1	0.15	5
Bed slope (current direction) (rad)	−0.01	0.7	2
Bed slope (perpendicular to current direction) (rad)	−0.02	0.7	2

The first value, spacing, and the number of points in each axis define the range of each condition that may appear during a simulation and influence sediment transport rates. The first value is the minimum value. The second value in each axis, except grain size, is the “First value $+$ Spacing” and so forth. The second value for grain size is the “First value $\times$ Spacing” and so on.

#### Representing groynes

A sub-grid approach, as outlined in DHI^61^, is used to account for the groynes presented at the test site in littoral drift simulations since the size of these are smaller than the mesh elements. Specifically, all groynes are defined as polylines. The nodes that make up these polylines have $x,y$ coordinates, which specify the location and geometry of the groyne, and an elevation ($z$) value relative to MHW.

#### MIKE21 SM

The following paragraphs provide extensive details on the parameterisation and computational framework of MIKE21 SM as this is the primary module that forms the basis for the continued development of the hybrid 2D/one-line modelling approach in this paper.

**Figure 6:**
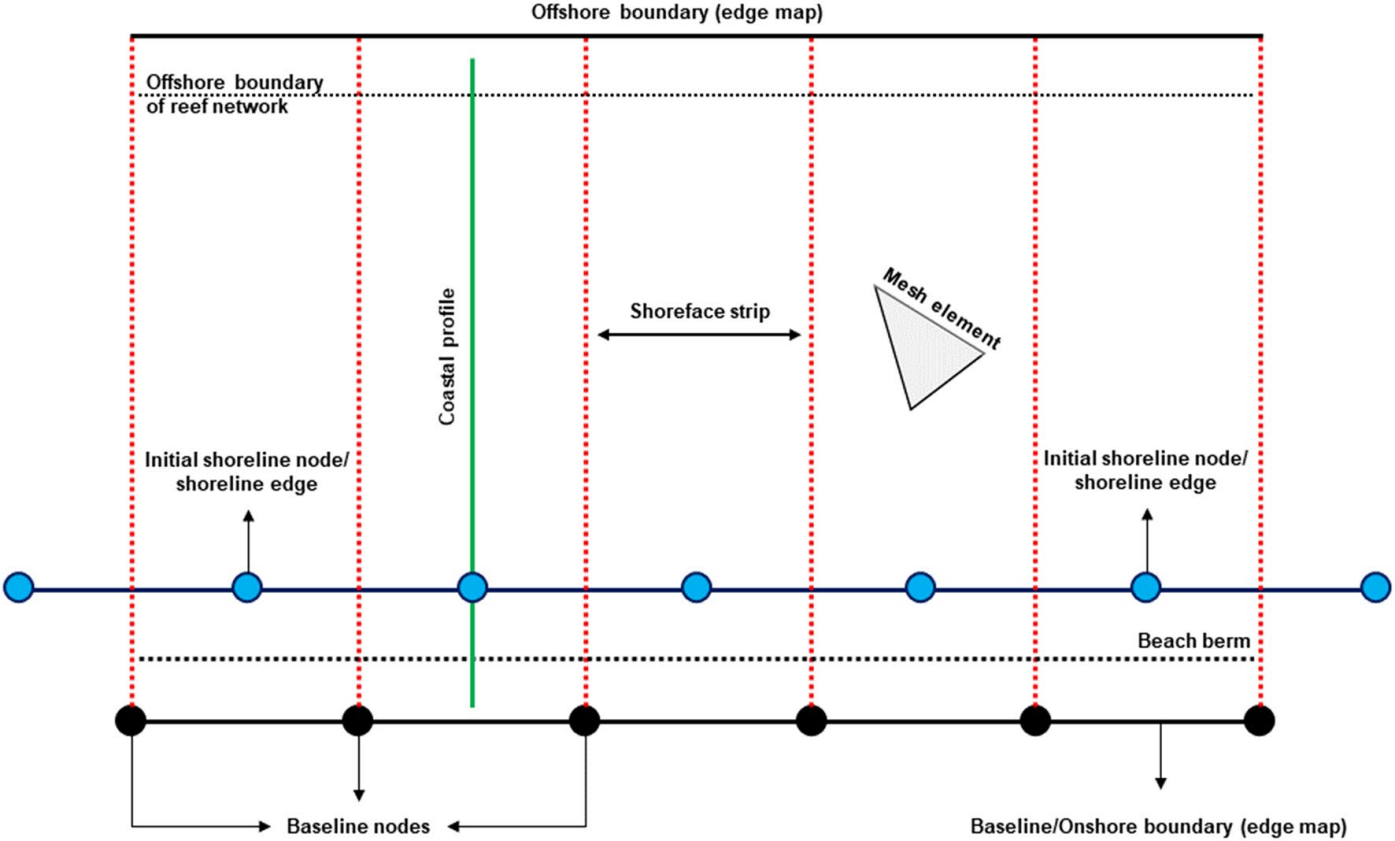
Computational setup of MIKE21 SM. MIKE21 SM uses an edge map that divides the shoreface into strips. Each strip has one active coastal profile and one shoreline edge. The active coastal profile in each shoreface strip moves with the shoreline edge perpendicular to the baseline, based on the total change in sediment volume within the strip. The spacings between two baseline nodes determine the resolution of the initial shoreline and longshore width of each shoreface strip. The onshore boundary of the edge map is the baseline, whereas the offshore boundary is the depth contour seaward of the closure depth.

MIKE21 SM requires four inputs to update the shoreface morphology: a baseline, an initial shoreline, an edge map, and predefined coastal profiles. The baseline and edge map define the spatial domain for MIKE21 SM calculations. The initial shoreline and predefined coastal profiles define the bathymetry inside this domain. Figure 6 shows the general configuration of MIKE21 SM domain.

The orientation of the baseline specifies the direction of shoreline movement in a simulation, and the initial shoreline defines the initial shoreline position that is subject to accretion and erosion. The baseline is landward of the beach berm, which forms the onshore extent of the active coastal profile. The beach berm elevation at the test site is 1.14 m above MHW. The baseline and initial shoreline are defined as polylines made up of nodes, each containing $x,y$ coordinates. The spacing between baseline nodes defines the resolution of the initial shoreline. The baseline and initial shoreline nodes are staggered, such that there is one shoreline node between two baseline nodes (Fig. 6). Each node in the initial shoreline defines one shoreline edge that moves shore-normal in a simulation. The baseline and initial shoreline have a resolution (nodal spacing) of 15 m.

The edge map assigns mesh elements to a shoreline edge by dividing the shoreface into strips perpendicular to the baseline. The baseline forms the onshore boundary of the edge map, and the resolution of the baseline defines the longshore width of each strip. Two baseline nodes generate one strip. Each strip has one shoreline node (shoreline edge) between baseline nodes (Fig. 6). The offshore boundary of the edge map is specified as the depth contour seaward of the closure depth, which is 5.8 m below MHW at the test site, to ensure that the edge map encompasses the cross-shore extent of the active coastal profile (beach berm to closure depth).

The coastal profile defines a representative cross-shore profile that moves shore-normal with a shoreline edge in a simulation. One coastal profile is specified in each shoreface strip as a vector polyline that runs through the initial shoreline node in the strip, perpendicular to the baseline (Fig. 6). Each coastal profile polyline extends beyond the beach berm and closure depth and comprises nodes with $x,y$ coordinates and an elevation ($z$) value relative to MHW. $z$ is interpolated from the mesh bathymetry. Each coastal profile polyline has a nodal spacing (resolution) of 1 m. Changes in sediment volume within the active coastal profile in a shoreface strip causes the shoreline edge (along with the profile) in the strip to move shore-normal during a simulation (Fig. 6). MIKE21 SM calculates the total sediment volume change in a shoreface strip by adding up the sediment volume change within each mesh element in the strip. Essentially, MIKE21 SM collapses the littoral drift gradients from MIKE21 ST onto the edge map, where it becomes integrated with the predefined active coastal profiles. In each shoreface strip, the active coastal profile moves seaward (landward) from sediment gain (loss) (Fig. 3).

### Model calibration

Following established guidelines and principles for calibrating coastal geomorphology models^74,75^, a stepwise approach is used to calibrate MIKE21 (i.e. tuning one parameter at a time) over a 2-year period (2014–2016) against six parameters based on the range of specifications in Table 5:

- *Nearshore spatial discretisation*, which defines the nearshore resolution in the mesh. The nearshore spatial discretisation affects the representation of the nearshore bathymetry, which influences the wave-current conditions that determine littoral drift gradients^6,51^. Littoral drift gradients are the primary driving flux of shoreline evolution in MIKE21^61^.
- *Bed resistance* based on Manning’s $n$ reciprocal (m^1/3^/s), which describes the friction acting on the flow as it moves over the mesh bathymetry^61^. The bed resistance affects the flow rate and wave dissipation, influencing sediment transport and redistribution over the mesh bathymetry^6,51,61^.
- *Sand porosity*, which affects the shoreface morphology by influencing the concentration of suspended sediments in the coastal system discretised within the model domain^51,61^.
- *Sand grain diameter (mm)*, which influences the mobility rate of sediments and the volume of sediment entrained in the flow. Littoral drift decreases as grain diameter increases^51^.
- *Sediment grading coefficient*, which describes the degree of sediment sorting in the coastal system discretised within the model domain, influencing littoral drift. Well-sorted sediments tend to have smaller grains and are less resistant to flow than poorly sorted sediments^51^.
- *Weir coefficient (m*^*1/2*^*/s)* of hard defences, which controls the overtopping discharge at hard defences, affecting sediment redistribution and flow around these structures^61,80^.

**Table 5:** Calibration parameters, inputs, and results.

Parameter	Units	Established range	Source	Values tested	MNC	MAC	BSS
					Obs: −0.01 m	Obs: 1.16 m	
Nearshore discretisation	M	N/A	N/A	**25**	−0.15	0.87	0.36
				30	−0.19	0.87	0.36
				35	−0.18	0.88	0.39
				40	−0.17	0.89	0.33
				45**	−0.17	0.88	0.38
				50	−0.19	0.96	0.24
				55	−0.18	1.01	0.15
				60	−0.2	1.17	−0.02
				65	−0.21	1.11	0.03
Manning’s n (Sandy beaches)	m^1/3/^s	Reciprocals: 29–50	^76^	29**	−0.17	0.83	0.41
				**32**	−0.17	0.88	0.38
				33	−0.17	0.89	0.38
				40	−0.17	1.16	0.01
				50	−0.21	1.26	−0.05
Sand grain diameter	Mm	0.0625–0.125 (very fine)	^78^	0.1	0.46	11.85	−73.66
		0.0125–0.25 (fine)		0.2**^a^	−0.17	0.79	0.44
		0.25–0.5 (medium)		0.25	−0.16	0.75	0.46
		0.5–1 (coarse)		0.5	−0.17	0.66	0.49
		1–2 (very coarse)		1	−0.19	0.62	0.51
Sand porosity	N/A	0.3 – 0.7	^77^	0.3**	−0.17	0.79	0.44
				**0.4**	−0.17	0.83	0.41
				0.5	−0.17	0.89	0.37
				0.7	−0.16	1.19	0.1
Sediment grading coefficient	N/A	< 1.27 (very well sorted)	^79^	1.1**	−0.17	0.79	0.44
		1.27–1.4 (well sorted)		1.3	−0.15	0.97	0.31
		1.41–1.99 (moderately sorted)		1.5	−0.15	1.41	−0.21
		2–3.99 (poorly sorted)		2	1.71	43.33	−1 169.67
		4–15.99 (very poorly sorted)					
		≥ 16 (extremely poorly sorted)					
Weir coefficient	m^1/2^/s	0.11–0.27 (lateral structure)	^80^	0.11	−0.16	0.76	0.46
		0.3–1.71 (broad crested structure)		0.55	−0.16	0.76	0.46
		1.77–2.26 (Ogee crested structure)		0.77	−0.16	0.76	0.46
		1.71–1.82 (sharp crested structure)		0.99	−0.16	0.76	0.46
				1.21**	−0.16	0.76	0.46
				1.44	−0.17	0.77	0.45
				1.82	−0.17	0.79	0.44
				**1.838**	−0.17	0.79	0.44
				2.21	−0.16	0.82	0.41

Bold values are the initial values. Values with two asterisks (**) are the values used after calibration.

*MNC* is mean net shoreline change, *MAC* is mean absolute shoreline change, *BSS* is Brier Skill Score, *Obs* is observed.

^a^0.2 mm sand grain diameter selected as this is more representative of observed sand properties at the test site.

The nearshore spatial discretisation is first calibrated to identify the independent mesh discretisation, defined as the mesh with the coarsest nearshore resolution that does not significantly affect model predictions. Identifying the independent mesh discretisation is important for ensuring that model predictions are due to the underlying physics solved in the model and not due to the mesh specifications. After identifying the independent mesh discretisation, bed friction is calibrated to identify the most optimal Manning’s $n$ reciprocal for subsequent simulations followed by sand porosity, sand grain diameter, sediment grading coefficient and the weir coefficient of hard defences. A stepwise calibration approach, as described here, is particularly useful for identifying the key parameters that cause the largest errors in shoreline evolution predictions. This knowledge is necessary for understanding (and refining) the intrinsic behaviour of shoreline evolution models^74,75^. The effects of the stepwise calibration process on shoreline evolution predictions are quantified using the Brier Skill Score (details in “Model verification”), and the results (Table 5) are used to identify the most optimal specifications for subsequent shoreline evolution simulations.

Calibration, in its traditional sense, is often limited to free parameters whose values are difficult to determine a priori or measure in the field^74,75,81,82^. Examples of these free parameters include bed friction, nearshore discretisation, and the weir coefficient of hard structures. The sediment parameters (grain size, porosity, grading, etc.) included in the model calibration process do not necessarily fall within this class of free parameters primarily because their specification in coastal models can be informed by field surveys or observed data^75^. The challenge here is that coastal sediment properties (grain size, porosity, grading) generally vary spatially and temporally^83,84^. As a result, it is difficult to define optimal specifications of these properties in shoreline evolution models, even if associated field data exist, as these models generally require a single value for each sediment parameter that broadly characterises the geomorphology of the coastal system being simulated (c.f.^61,73,85,86^). This is a particular challenge for parameterising meso timescale shoreline evolution models since there is often considerable temporal and spatial variability in sediment properties over these timescales. Also, temporal and spatial data on sediment properties are rarely available, which makes specifying these properties in a model even more challenging^75^. For these reasons, sediment parameters are now widely incorporated in the calibration of shoreline evolution models^74,87–90^. Caution, however, is needed here to ensure that the calibration process is bounded by physically realistic estimates of all calibration parameters otherwise there is the risk of erroneously discretising observed coastal morphology in the model domain, which can compromise the reliability of resulting shoreline evolution predictions^75^. Therefore, in this study, the calibration of sand grain diameter, porosity, and grading coefficient (a) are bounded by physically realistic ranges established for these parameters^77–79^ and (b) considers recorded values of sediment properties obtained from historical field surveys at the test site^64^. The calibration of (a) nearshore discretisation is bounded by the spatial scales over which primary drivers of shoreline evolution operate (Table 1), (b) bed resistance is bounded by physically realistic estimates of Manning’s $n$ reciprocal established for sandy coastal systems^76^, and (c) the weir coefficient of hard defences is bounded by physically realistic ranges established for groyne structures that are similar to those present in the test site^80^.

### Enabling a time-varying closure depth

**Figure 7:**
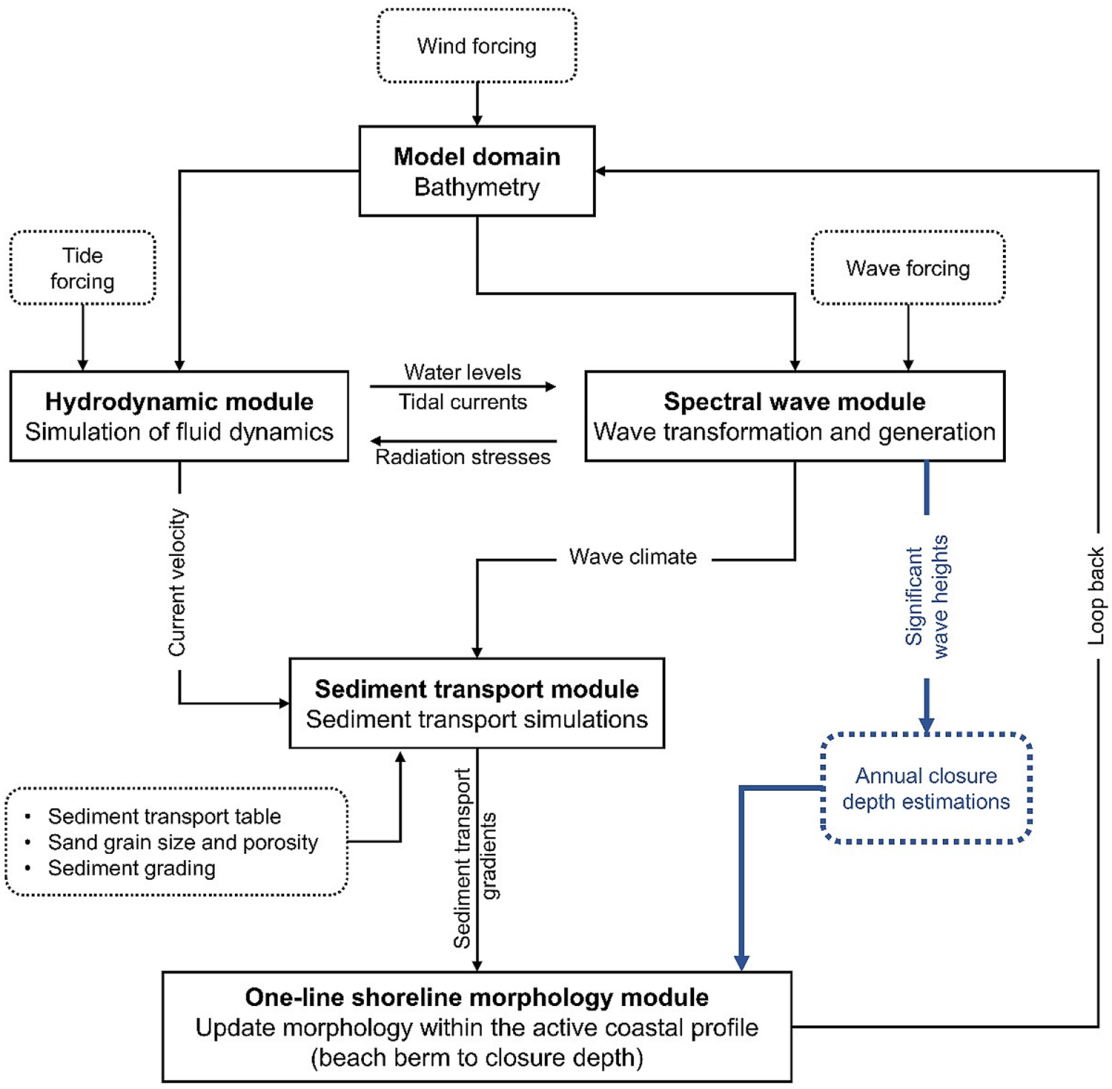
Schematic illustration of the modified hybrid 2D/one-line approach developed and tested. Comparison with Fig. 3 shows that the key aspect of the hybrid 2D/one-line approach modified is the introduction of annual variations in the closure depth, which are calculated from the wave heights data generated by the spectral wave module, within the one-line shoreline morphology module. This modification allows us to account for the effects of sea-level rise on the coastal profile in the one-line shoreline morphology update. All modifications made are highlighted in blue.

The approach developed to incorporate a time-varying closure depth entails running MIKE21 annually with a different closure depth (based on nearshore significant wave heights) and using the morphology (mesh bathymetry) and hydrodynamic (wave, flow, and sediment transport) outputs from each annual simulation to run subsequent annual simulations, as illustrated in Fig. 7. This approach does not modify any code within MIKE21. Instead, it forces MIKE21 to simulate shoreline evolution iteratively over one-year periods, which incorporate a change in the closure depth. Albeit manually forced, this iterative procedure allows the offshore part of the active coastal profile to vary vertically over time based on the changing hydrodynamic conditions in the model domain. In theory, this approach enables us to account for sea-level and wave climate variations, both of which influence the evolution of coastal profiles and associated shoreline morphodynamics^18,47^. A time-varying closure depth is also likely a plausible solution for reducing the gross simplifications of the one-line theory assumptions that underpin the shoreline morphology update in hybrid 2D/one-line models, which forces the coastal profile to maintain fixed vertical limits contrary to the underlying physics of coastal profile evolution.

To evaluate the above approach, four meso timescale hindcast simulations of shoreline evolution (1966–2016) are carried out in the test site, as outlined below. Hereafter, these simulations are referred to as Models A to D. Models A and B apply the one-line theory principles to update the shoreline morphology. These models provide baseline predictions for testing whether a time-varying closure depth improves meso timescale shoreline evolution predictions. Model C generates boundary conditions data for model D, which accounts for temporal variations in the closure depth.

#### Model A

This model maintains the one-line theory principles of the traditional hybrid 2D/one-line approach, assuming a temporally constant closure depth in the shoreline morphology update. It is applied using the independent mesh discretisation, identified from calibration, interpolated with the 1966 DEM obtained. The closure depth is 4.2 m, which is the most seaward contour mirroring the shape of the shoreline in the 1966 DEM. Other specifications include the calibrated values of free parameters considered in “Model calibration”. All other parameters and boundary conditions are specified as outlined in “Model parameterisation” and Tables 3 and 4.

#### Model B

**Figure 8:**
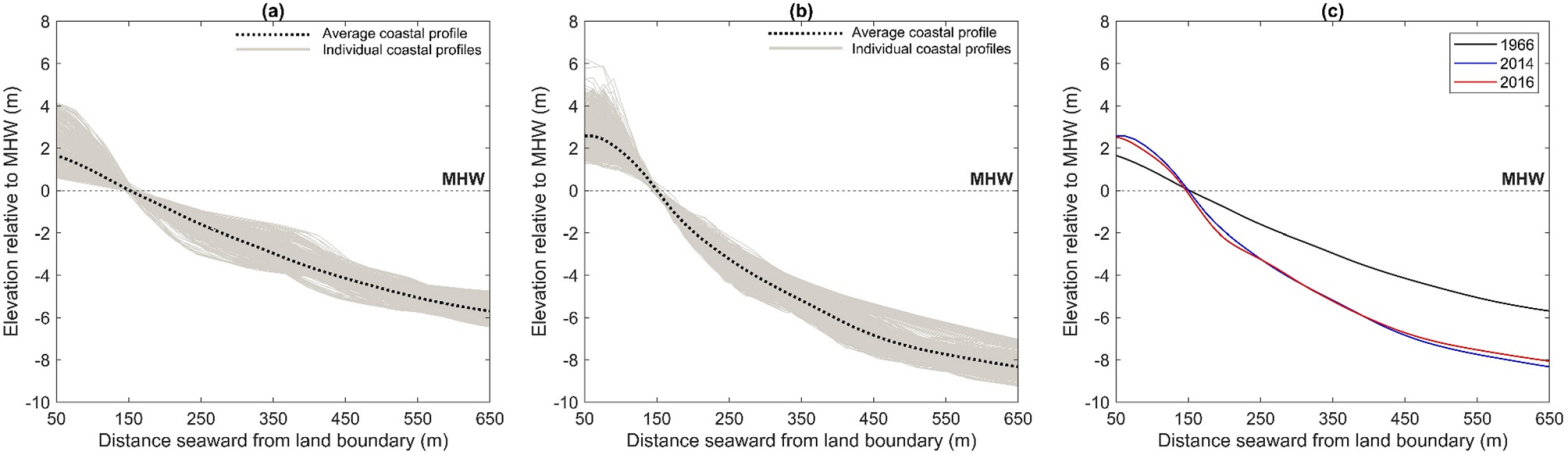
Temporal comparison of the average coastal profile morphology at the test site. (**a**) The 1966 coastal profile envelope and average coastal profile. (**b**) The 2014 coastal profile envelope and average coastal profile. (**c**) Compares the 1966, 2014, and 2016 average coastal profiles. The average coastal profile is the average of individual cross-shore profiles sampled every 15 m longshore. The individual cross-shore profiles incorporated in (**a,b**) illustrate the coastal profile envelope.

This model *fully* enforces the one-line theory assumptions and has the same setup as model A, except mesh bathymetry and closure depth. It is applied using the independent mesh discretisation interpolated with a modified DEM. The modified DEM is created by interpolating the 1966 DEM data points with the depth ($z$) values from the 2014 DEM. In principle, this interpolation shifts the 2014 coastal profiles back to their 1966 position in line with the one-line theory. The one-line theory assumes that coastal profiles keep a constant time-averaged form whilst moving shore-normal from a change in sediment balance. The 1966 and 2014 coastal profiles are notably different (Fig. 8). MIKE21 and related hybrid 2D/one-line models do not simulate the three-dimensional physics of coastal profile evolution. Instead, these models work on the premise that the active coastal profile maintains an equilibrium form. Therefore, using the 1966 DEM to simulate shoreline evolution through to 2016 may compromise the accuracy of the hybrid 2D/one-line approach. Shifting the 2014 coastal profiles back to their 1966 position will ensure the initial (1966) and predicted (2016) profiles have a similar shape to uphold the one-line theory principles that underpin the shoreline morphology update in the hybrid 2D/one-line approach. The 2014 and 2016 coastal profiles have negligible differences (Fig. 8). Using the 2014 coastal profile shape is thus appropriate for maintaining the one-line theory principles. The closure depth in this model is 6 m, which is the most seaward contour mirroring the shape of the shoreline in the modified DEM.

#### Model C

This model updates the mesh bathymetry annually to facilitate model D. MIKE21 provides the updated mesh bathymetry at each output time-step as an interpolated mesh, which can form the computational basis for subsequent simulations^61^. However, MIKE21 only updates the mesh bathymetry between the beach berm and closure depth contours. Model C has the same setup as model B, except the most seaward depth contour in the initial mesh bathymetry (i.e. the mesh bathymetry at the start of the simulation) is specified as the closure depth. Doing so ensures that MIKE21 updates the bathymetry in the entire mesh at each output time-step. The updated mesh bathymetry generated at each output time step ensures that there is an appropriate time-stamped mesh bathymetry to simulate shoreline evolution iteratively over one-year intervals in order to facilitate a time-varying closure depth in model D.

#### Model D

This model iteratively simulates shoreline evolution over one-year intervals, with each interval having a revised closure depth. Specifically, it executes 50 annual hindcast simulations from 1966 to 2016. The first annual hindcast (1966–1967) has the same setup as model B. Each subsequent annual hindcast (i.e. 1967–1968; 1969–1970; 1971–1972, etc.) has the same setup as the first one (1966–1967) except: (a) the relevant time-stamped mesh bathymetry output from model C is used as the computational basis (e.g. the 1967 mesh bathymetry output from model C is used to carry out the second annual hindcast, which runs from 1967 to 1968), and (b) closure depth. The closure depth in the second annual hindcast and each thereafter is calculated using the nearshore significant wave heights generated in the model domain from the preceding annual hindcast based on Birkemeier^91^ formula:

2 $${D}_{c}=1.57{H}_{e}$$

where ${H}_{e}$ is the effective wave height defined as:

3 $${H}_{e}= {\overline{H} }_{s}+5.6{\sigma }_{s}$$

where ${\overline{H} }_{s}$ is the annual mean significant wave height, and ${\sigma }_{s}$ is the associated standard deviation. The significant wave heights generated in the nearshore are used to calculate closure depths because this is where refraction and shoaling modify the wave characteristics that affect shoreline evolution^92^. The calculated shoreline position and hydrodynamic fields (wave, current, and sediment transport) from one annual hindcast are used to run the next annual hindcast. For example, the calculated shoreline position and hydrodynamic fields from the first annual hindcast are used to run the second annual hindcast and those from the second annual hindcast are used to run the third annual hindcast and so on. The mesh bathymetry and the closure depth are the only inputs updated annually in model D. Table 6 provides the closure depth estimated and applied in each annual hindcast.

**Table 6:** Closure depth time series estimates used to hindcast meso timescale shoreline evolution (1966–2016) compared against associated observations.

Year	Estimated (m below MHW)	Observed (m below MHW)	Net difference (m)
**1966**	**6**	**No observed closure depth data**	
**1967**	**7.93**		
**1968**	**5.45**		
**1969**	**7.82**		
**1970**	**5.8**		
**1971**	**7.39**		
**1972**	**6.36**		
**1973**	**7.4**		
**1974**	**6.24**		
**1975**	**7.32**		
**1976**	**6.09**		
**1977**	**7.18**		
**1978**	**6.42**		
**1979**	**7.29**		
1980	6.55	6.67	−0.12
*1981*	*7.29*	*6.96*	*0.34*
*1982*	*6.85*	*6.31*	*0.54*
*1983*	*7.22*	*7*	*0.22*
1984	6.98	8.21	−1.23
*1985*	*6.95*	*6.67*	*0.28*
*1986*	*7.15*	*6.61*	*0.54*
*1987*	*6.78*	*5.45*	*1.33*
*1988*	*7.24*	*5.7*	*1.54*
*1989*	*6.19*	*5.53*	*0.66*
*1990*	*7.66*	*4.43*	*3.23*
1991	5.23	6.41	−1.18
1992	7.93	8.76	−0.83
1993	5.45	7.99	−2.54
*1994*	*7.74*	*7.25*	*0.48*
1995	5.89	6.47	−0.58
1996	7.48	7.9	−0.41
*1997*	*6.27*	*5.2*	*1.08*
*1998*	*7.4*	*6.41*	*1*
*1999*	*6.23*	*6.08*	*0.15*
*2000*	*7.4*	*5.62*	*1.78*
*2001*	*6.09*	*5.93*	*0.16*
*2002*	*7.28*	*5.06*	*2.23*
2003	6.43	8.2	−1.76
*2004*	*7.29*	*5.31*	*1.99*
2005	6.55	6.58	−0.03
*2006*	*7.29*	*7.17*	*0.12*
*2007*	*6.85*	*6.56*	*0.29*
*2008*	*7.21*	*7.17*	*0.03*
2009	7	8.93	−1.93
2010	6.95	8.32	−1.37
2011	7.25	7.74	−0.49
2012	6.78	10.49	−3.71
**2013**	**7.24**	**No observed closure depth data**	
**2014**	**6.27**		
**2015**	**7.75**		

Net difference (m) $=$ estimated $-$ observed closure depth.

Bold rows indicate non-verifiable closure depth estimations, italics rows indicate closure depth overestimation, and non-highlighted rows indicate closure depth underestimation.

The iterative process adopted in model D, which is summarised in Fig. 7, can be easily extended to any shoreline evolution model that applies the one-line theory in the shoreline morphology update. Although Birkemeier^91^ formula is used for the closure depth estimation, the simple iterative process adopted in model D can be modified to accommodate alternative closure depth estimation methods, such as those reviewed in Valiente et al.^71^. Birkemeier formula is selected in this study because the original Hallermeier^93^ closure depth formula over-predicts the closure depth by approximately 25%^71,94^. However, the closure depth equation applied is non-consequential to the overarching purpose of this study, which seeks to determine whether we can obtain more theoretically realistic predictions of meso timescale shoreline evolution under sea-level rise from incorporating a time-varying closure depth in hybrid 2D/one-line models.

To determine whether a time-varying closure depth improves meso timescale shoreline evolution predictions, the accuracy of models A, B, and D are quantified using the methods in “Model verification”.

### Model verification

2449 cross-shore transects, spaced every 5 m longshore, are generated and used to quantify shoreline evolution observations and predictions over the 50-year hindcast period. The data obtained are applied to calculate the accuracy of shoreline evolution predictions using the Brier Skill Score ($BSS$):

4 $$BSS=1- \frac{\sum {\left({Sh}_{obs}- {Sh}_{pred}\right)}^{2}}{\sum {\left({Sh}_{obs}- {Sh}_{init}\right)}^{2}}$$

where ${Sh}_{int}$ is the initial shoreline position per transect (i.e. the shoreline position observed at the start of the simulation), ${Sh}_{pred}$ is the predicted shoreline position per transect (i.e. the shoreline position predicted at the end of the simulation), and ${Sh}_{obs}$ is the observed shoreline position per transect (i.e. the shoreline position observed at the end of the simulation). $BSS$ ranges from −∞ to 1. A $BSS$ of 1 indicates perfect agreement between ${Sh}_{obs}$ and ${Sh}_{pred}$, 0 indicates that ${Sh}_{pred}$ is closer to ${Sh}_{init}$, and a negative $BSS$ indicates that ${Sh}_{pred}$ is further away from ${Sh}_{obs}$. The $BSS$ classification scheme developed by Sutherland et al.^95^, which considers a score of 1–0.5 as excellent, 0.5–0.2 as good, 0.2–0.1 as reasonable, 0.1–0 as poor, and ≤ 0 as bad, is adopted in this paper.

Descriptive statistics, measures of average error, and spatial line plots are also used to quantify and visualise the differences in the longshore trend between shoreline evolution observations and predictions. The information from these is used to better interpret all $BSS$ estimations.

## Results and analysis

**Figure 9:**
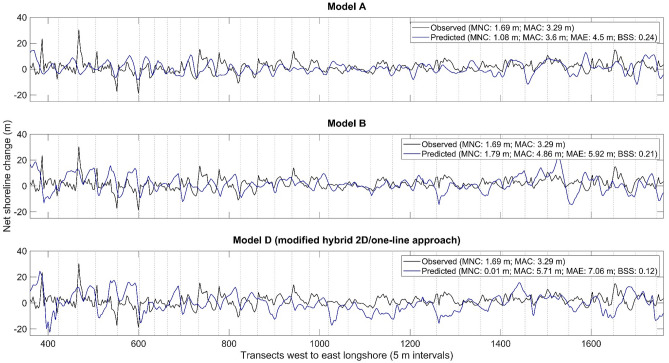
Observations and predictions of shoreline evolution from models A, B, and D (1966–2016). Model A applies the traditional hybrid 2D/one-line approach, assuming a constant closure depth (4.2 m). Model B also adopts the traditional hybrid 2D/one-line approach (closure depth = 6 m) but applies the one-line theory principles in both sediment transport simulations and shoreline morphology update. Model D applies the modified hybrid 2D/one-line approach developed, incorporating temporal variations in the closure depth (range  5.23–7.93 m). MNC is mean net change, MAC is mean absolute change, MAE is mean absolute error, and BSS is Brier Skill Score. Grey vertical dashed lines indicate groyne locations.

We can see a relatively good fit between shoreline evolution observations and predictions from models A and B over the 50-year hindcast (Fig. 9a,b). The key difference between both models is that model A only applies the one-line theory in the shoreline morphology update whereas model B applies the one-line theory in both littoral drift simulations (through the mesh bathymetry) and shoreline morphology update. Shoreline evolution predictions from models A and B have a borderline good $BSS$ of ~ 0.2 (Fig. 9a,b), indicating that these predictions generally move in the same direction as the corresponding observations. Movements in the same direction are a good sign that a model is able to replicate observed phenomena. The wider implications of these findings is that the one-line theory, as included in the hybrid 2D/one-line approach, provides relatively good meso timescale shoreline evolution predictions^95^. Models A and B borderline good $BSS$ s correspond to associated shoreline evolution observations and predictions demonstrating:

- Net shoreline accretion, evident from the positive mean net shoreline change (MNC) observed (1.69 m; standard deviation = 4.16) and predicted from models A (1.08 m; standard deviation = 4.45) and B (1.79 m; standard deviation = 6.14) over the 50-year hindcast (Fig. 9a,b). In terms of longshore trends in shoreline morphology, shoreline evolution observations (67% accretion; 33% erosion) and predictions from models A (60% accretion; 40% erosion) and B (62% accretion; 38% erosion) all indicate that accretion dominated ≥ 60% of the test site over the 50-year hindcast period. The longshore range in shoreline evolution observations (−18.8 to 30.2 m) and predictions from models A (−11.9 to 14.6 m) and B (−14.4 to 23.1 m) also show higher accretion magnitudes than erosion magnitudes over the 50-year hindcast.
- A similar longshore pattern of accretion and erosion (Fig. 9a,b). Shoreline evolution observations and predictions from models A and B all show an alternating pattern of accretion and erosion over the 50-year hindcast, with accretion mainly between groynes and erosion mostly in the immediate vicinity of groynes (Fig. 9a,b). This alternating pattern is common in sandy coastal systems that have groyne fields for intercepting littoral drift in order to facilitate shoreline stabilisation and expansion of beach width^96,97^.

The net accretion observed over the 50-year hindcast period is primarily attributed to the groynes constructed between 1930 and 1961 at the test site^62–64^. The groynes were used to offset the effects of sediment deficit and sea-level rise^63,64^. Before the construction of groynes, the shoreline at the test site was in a state of natural retreat^63,98^. Models A and B ability to predict the correct net shoreline change trend and general alternating pattern of accretion and erosion observed from 1966 to 2016 in the test site, therefore, indicate that the hybrid 2D/one-line approach can successfully simulate the effects of groynes on shoreline evolution under sea-level rise. Groynes are incorporated in both models as subgrid polyline features, as outlined in “Representing groynes”.

Models A and B assumption of a constant closure depth in accordance with the one-line theory is the most plausible reason that prohibited a better fit between corresponding shoreline evolution observations and predictions^51^. The assumption of a constant closure depth in models A and B averages out the temporal variability of the active coastal area at the test site over the 50-year hindcast (Table 6). Data from 1980 to 2012 show that the closure depth varies annually in the test site (Table 6)^99^. There are no closure depth data before and after this period. The closure depth defines the offshore extent of morphodynamic activity in hybrid 2D/one-line models, influencing the horizontal dimension (cross-shore width/extent) of the active coastal area over which littoral drift becomes uniformly distributed and cause changes in shoreline movement. Therefore, the achievable goodness of fit between shoreline evolution predictions and observations from using a constant closure depth in hybrid 2D/one-line models will likely reduce as (a) timescales increase and (b) the active coastal area changes. This became apparent from the calibrated hybrid 2D/one-line model applied through MIKE21 (2014–2016) having a good $BSS$ of 0.46 (Table 5) whereas models A and B (1966–2016) have a borderline good $BSS$ (~ 0.2). Models A and B are meso timescale extensions of the calibrated model. Closure depth changes typically occur in response to a change in wave climate or sea-level^45,46^. Thus, the use of a constant closure depth means that the effects of sea-level rise over the 50-year hindcast are not fully incorporated in models A and B. However, the 1966–2016 tide gauge data (Table 2) forced at the sea boundary in the mesh ensures that models A and B account for sea-level rise in littoral drift simulations. The challenge here is accounting for the effects of sea-level rise in the shoreline morphology update. A potential solution for this challenge is to allow the closure depth to vary over time in the shoreline morphology update in response to changing hydrodynamics in the model domain^51^.

Results show that shoreline evolution predictions worsen in response to the time-varying closure depth specified over the 50-year hindcast (Fig. 9c). This is evident from model D having a $BSS$ of 0.12 (reasonable) whereas models A and B (constant closure depth) have a $BSS$ slightly above 0.2 (borderline good) (Fig. 9). The reasonable $BSS$ from model C corresponds to associated shoreline evolution predictions (MNC = 0.01 m) and observations (MNC = 1.69 m) showing net accretion and a fairly similar alternating pattern of accretion and erosion, with accretion mainly between groynes and erosion mainly in the direct vicinity of groynes (Fig. 9c). These consistencies between shoreline evolution observed and predicted from model D provide further evidence that the hybrid 2D/one-line approach can successfully simulate the effects of groynes on shoreline evolution under sea-level rise. However, the longshore trends in the shoreline evolution predictions from model D (51% accretion; 49% erosion) show more erosion than observed (67% accretion; 33% erosion) and predicted from models A (60% accretion; 40% erosion) and B (62% accretion; 38% erosion), which explain the corresponding decline in $BSS$ (Fig. 9).

Model D overprediction of shoreline erosion corresponds to net closure depth overestimation from 1980 to 2012 (Table 6). Closure depth overestimations are evident from 1981 to 1990 (range = 0.22–3.23 m) and from 1997 to 2008 (range = 0.03–2.23 m). Table 6 also shows closure depth underestimations from 1991 to 1996 (range = 0.41–2.54 m) and from 2009 to 2012 (range = 0.49–3.71 m). In MIKE21 and related hybrid models, closure depth overestimations push the observed seaward extent of the active coastal profile further offshore, causing sediment distribution in morphologically inactive areas^51,100^. Closure depth overestimations consequently reduce the volume of sediment available for inshore distribution, which causes an overprediction (underprediction) of shoreline erosion (accretion)^51,101^. This finding is apparent from comparing the shoreline evolution predictions from models B and D (Fig. 9b,c). Model D predicts more erosion than model B (Fig. 9b,c) since it has a mean deeper closure depth (range = 5.23–7.93 m; mean = 6.86 m; standard deviation = 0.68) than model B (6 m). All other inputs in models B and D are the same. Shoreline evolution predictions in response to the time-varying closure depth specified in model D are, therefore, within realistic expectations.

**Figure 10:**
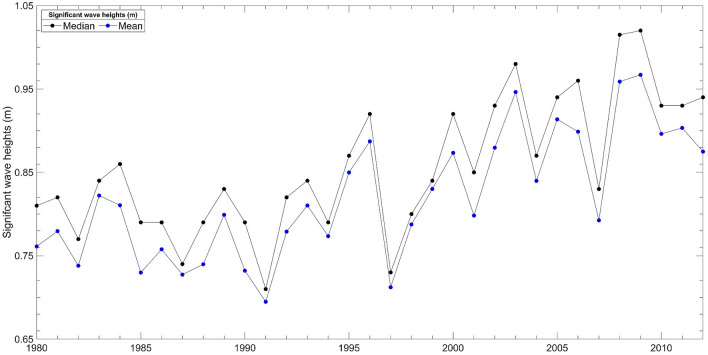
Annual median and mean significant wave height statistics (1980–2012) recorded at the test site.

All closure depth estimations applied in model D are derived from Birkemeier^91^ formula using nearshore significant wave heights calculated in the model domain. Nearshore significant wave heights operating in the model domain are influenced by the boundary conditions forced in the model, including mesh bathymetry, tides, wind, and wave climate. Closure depth overestimations in model D are most plausibly a consequence of extending the 2014–2016 wave climate data obtained (Table 2) over the 50-year hindcast. Figure 10 illustrates an increasing trend observed in the annual median and mean significant wave height from 1980 to 2012 at the test site^99^. There are no wave climate statistics before and after this period. Considering the increase in significant wave heights from 1980 to 2012, extending the 2014–2016 wave climate data in model D most plausibly caused MIKE21 to overestimate significant wave heights over the 50-year hindcast, which can explain the overestimated closure depths.

Considering wave climate data limitations and related closure depth overestimations, the reasonable fit between model D predictions and associated observations imply that enabling a time-varying closure depth in hybrid 2D/one-line models *may* improve meso timescale shoreline evolution predictions if closure depths can be accurately prescribed over time. A time-varying closure depth will enable us to explicitly account for sea-level rise in meso timescale shoreline evolution predictions, which so far has only been possible in the Bruun Rule^102^. A key effect of sea-level rise is temporal changes in the closure depth^45,103^. However, obtaining physically realistic time series estimates of closure depths depend on the availability of high-quality wave climate data or predictions. In the absence of high-quality wave climate data or predictions, estimating and applying a time-varying closure depth in hybrid 2D/one-line models will generate greater uncertainty in meso timescale shoreline evolution predictions relative to using a constant closure depth (Fig. 9). Verifiable meso timescale wave climate and closure depth data are needed to better evaluate whether a time-varying closure depth can significantly improve shoreline evolution predictions over these timescales. As it stands, a time-varying closure depth offers the most plausible solution to account for sea-level rise in shoreline evolution predictions. This assertion is based on the fact that the closure depth changes as sea-level changes, which is not accounted for in hybrid 2D/one-line models due to their assumption of a constant closure depth in line with the one-line theory.

## Discussion

A wider implication of the results is that the equilibrium profile concept of the one-line theory appears to be valid for simulating meso timescale shoreline evolution. This is evident in the following instances:

- Models A and B having a borderline good $BSS$ of ~ 0.2. Model A applies the principles of the one-line theory only in the shoreline morphology update whereas model B applies the one-line theory principles in both the shoreline morphology update and littoral drift simulations. Importantly, both models were able to replicate observed shoreline evolution trends and patterns reasonably well.
- Model D has a reasonable $BSS$ of 0.12. Although model D varies the closure depth over time, it still maintains the one-line theory principles of an equilibrium coastal profile. Despite modifications to the equilibrium profile concept, model D predicts the overall net shoreline change trend observed in the test site.

A fundamental criticism of the equilibrium profile concept is the assumption of a temporally constant closure depth, which will inevitably change under sea-level rise. A closer inspection of the results reveals that this assumption leads to a decline in shoreline evolution prediction accuracy as the timescale increase. For example, the $BSS$ of the calibrated hybrid 2D/one-line model (2014–2016) declined from 0.46 to ~ 0.2 when applied over the 50-year hindcast period through models A and B. However, we can argue that this decline in model accuracy with increasing timescale may be due to the wave climate and wind data obtained for the two-year model calibration being replicated over the 50-year hindcast period. Although this is a valid argument, the assumption of a temporally constant closure depth is invalid. Therefore, while we may obtain reasonable shoreline evolution predictions from applying this assumption, we need to be cautious that we are not obtaining a *right model* for the *wrong reasons* (i.e. running into the problem of equifinality)^104,105^. Right in this context refers to the best match between observed and predicted shoreline evolution and wrong refers to physically unrealistic input values of the conceptual model describing observed coastal morphology. Equifinality is an important issue to consider because it means that an optimal set of model inputs may appear to provide good predictions of the variable of interest (shoreline position in this case) but actually provide poor predictions of variables that are not considered in model calibration and/or validation (e.g. beach profiles in this case)^105^. It is possible that predicted variables not considered in model calibration and/or validation are used to inform coastal management decisions. Thus, we need to ensure that our model predictions are reflective of the underlying physics of coastal evolution and not just optimal model parameterisation otherwise we run the risk of misinforming coastal management^105^.

A key observation from the results is that the nearshore wave climate seems to have a dominant influence on meso timescale shoreline evolution in sandy coastal systems. This insight stems from the time-varying closure depth in model D, which is calculated in response to nearshore wave heights, significantly affecting shoreline evolution predictions (Fig. 9). Nearshore wave climate, which is indirectly influenced by sea-level rise, affects wave energy dissipation and the direction that waves approach the shoreline, determining the longshore flux of sediment that influence shoreline accretion and erosion patterns^106^. Sea-level rise can also directly influence shoreline evolution by forcing shorelines to retreat. However, sediments eroded due to shoreline retreat from sea-level rise typically become entrained in littoral drift, often getting trapped and deposited elsewhere along the shoreline, especially in managed sandy coastal systems^13,55,107^. Littoral drift can, therefore, cause segments of managed sandy shorelines to accrete under sea-level rise by increasing the sediment budget downdrift of defences^108^, as observed over the 50-year hindcast period in the test site (Fig. 9). Slott et al.^52^ show that the longshore variation in shoreline evolution rates from littoral drift can be an order of magnitude higher than those expected from sea-level rise alone. Nearshore wave climate generally changes over years to decades and are partly dependent on water depth due to shallow water effects on wave propagation^109^. Water depths, on the other hand, will change under sea-level rise. Sea-level rise, thus, indirectly affects the nearshore wave climate and associated littoral drift. In this regard, a good proxy of both wave climate and sea-level change is the closure depth, which marks the depth limit of significant wave action and cross-shore sediment transport^52,53^. As hybrid 2D/one-line models assume a constant closure depth in line with the one-line theory, their prediction accuracy will likely reduce as wave climate and sea-levels change over increasing timescales, which we see from comparing the calibrated and meso timescale hindcast results. Hence, we need to account for temporal variations in the nearshore wave climate and sea-level to reliably simulate meso timescale shoreline evolution, which is analogous to the findings of Nicholls et al.^35^, De Figueiredo et al.^101^, and Nguyen et al.^53^.

Accounting for the effects of temporal variations in nearshore wave climate and sea-level on meso timescale shoreline evolution predictions is challenging because this requires us to incorporate the three-dimensional effects of these variables in shoreline evolution models. This requirement is necessary since the nearshore wave climate and sea-level primarily affect shoreline morphology through their influence on coastal profile evolution, which is a complex three-dimensional process. The challenge here is that there is considerable uncertainty with respect to modelling the three-dimensionality of coastal processes (e.g. undertow currents) over meso timescales^25^.

A time varying closure depth appears to provide a promising solution to account for nearshore wave-climate and sea-level variations in meso timescale shoreline evolution predictions. This insight stems from the theoretically realistic meso timescale shoreline evolution predictions derived from applying a time-varying closure depth in model D (Fig. 9). A notable effect of nearshore wave climate and sea-level variations is a change in the depth limit of significant wave action (the closure depth), which affects the shape of the coastal profile and ensuing shoreline morphodynamics^50^. The key novelty of the new hybrid 2D/one-line approach developed and applied through model D is that specifying a time-varying closure depth (i.e. variations in the offshore *vertical* limit of the active coastal profile over time) in response to nearshore wave climate enables us to incorporate the three-dimensionality of coastal profile evolution whilst maintaining the principles of the one-line theory equilibrium profile concept. The equilibrium profile concept is needed to stabilise the shoreline morphology update, as previously discussed.

The coastal modelling literature still remains largely in a state of trial and error, experimenting with novel modelling approaches that can help us make better sense of coastal environments and their evolution over various time and space scales^110^. For instance, there has been a clear linear progression in the evolution of meso timescale shoreline evolution models since the study of Ashton et al.^111^, one of the first studies to apply the hybrid 2D/one-line modelling approach over meso timescales. Since then, this hybrid approach has evolved to include complex shoreline shapes, drift-dominated shorelines, high-angle wave instabilities, and shoreline stabilisation schemes^18,30,33,42,112^. The promising results from the new hybrid 2D/one-line approach presented in this paper can help facilitate the continued advancement of meso timescale shoreline evolution modelling approaches as these are becoming fundamentally important for informing local and regional scale coastal management and adaptation decisions^1,2^. In particular, further research is recommended to verify whether enabling a time-varying closure depth in hybrid 2D/one-line models can provide an effective and *physically realistic* approach for simulating meso timescale shoreline evolution under sea-level rise. After all, the reliability of meso timescale shoreline evolution predictions affect the credibility of associated coastal management decisions.

## Conclusion

Hybrid 2D/one-line shoreline models, which are becoming increasingly applied over meso timescales (10^1^–10^2^ years) to inform coastal management, cannot account for sea-level rise, an endogenous driving factor of shoreline evolution over these timescales. This inability stems from the one-line theory assumption of a spatially invariable closure depth, which underpins the shoreline morphology update in these models. Therefore, a new hybrid 2D/one-line approach was developed using MIKE21, which incorporates a time-varying closure depth in response to nearshore significant wave heights, a good proxy of sea-level change. This new hybrid approach was applied to hindcast shoreline evolution over a 50-year period along the Atlantic coast of Long Beach Barrier Island together with the standard hybrid approach, which assumes a constant closure depth. Results show that the time-varying closure depth specified caused an overprediction of shoreline erosion, attributed to net closure depth overestimation. Overestimating the closure depth forces sediment distribution in morphologically inactive areas offshore, resulting in either an overprediction of shoreline erosion or an underprediction of shoreline accretion. In this regard, results obtained from applying a time-varying closure depth are within expectations and, thus, promising. Hence, it is possible that developing hybrid 2D/one-line models to account for temporal variations in the closure depth may improve our ability to simulate meso timescale shoreline evolution under sea-level rise if closure depth estimations can be accurately prescribed over time. Further work is needed to verify this before a time-varying closure depth can be ruled out or accepted as a practical solution for incorporating sea-level rise effects in shoreline evolution models.

## Data Availability

All data used to inform this study are available from open-source repositories either hosted by the National Oceanic and Atmosphere Administration or the United States Geological Survey^56–60,99^. All data generated from this study are available from the corresponding author on reasonable request.
